# Perioperative management in gynecological surgery based on the ERAS program: Number 2 - February 2022

**DOI:** 10.1055/s-0042-1743401

**Published:** 2022-02-25

**Authors:** Aline Evangelista Santiago, Agnaldo Lopes da Silva Filho, Eduardo Batista Cândido, Paulo Ayrosa Ribeiro, Julio César Rosa e Silva, Walquíria Quida Salles Pereira Primo, Jesus Paula Carvalho, Sérgio Podgaec, Carlos Augusto Pires Costa Lino, Ricardo de Almeida Quintáiros, Luiz Gustavo Oliveira Brito

**Affiliations:** 1 Universidade Estadual Paulista “Júlio de Mesquita Filho”, Botucatu, SP, Brazil; 2 Universidade Federal de Minas Gerais, Belo Horizonte, MG, Brazil; 3 Universidade Federal de Minas Gerais, Belo Horizonte, MG, Brazil; 4 Faculdade de Ciências Médicas da Santa Casa de São Paulo, São Paulo, SP, Brazil; 5 Faculdade de Medicina de Ribeirão Preto, Universidade de São Paulo, Ribeirão Preto, SP, Brazil; 6 Universidade de Brasília, Brasília, DF, Brazil; 7 Faculdade de Medicina, Universidade de São Paulo, São Paulo, SP, Brazil; 8 Hospital Israelita Albert Einstein, São Paulo, SP, Brazil; 9 Hospital São Rafael, São Paulo, SP, Brazil; 10 Universidade do Estado do Pará, Belém, PA, Brazil; 11 Universidade Estadual de Campinas, Campinas, SP, Brazil

## Key points

- The Enhanced Recovery After Surgery (ERAS) program is based on perioperative medical optimization, including pre-admission counseling, pain relief, carbohydrate intake, thromboembolism prophylaxis, standard anesthetic protocol, optimized intraoperative fluid administration, recovery of normal gastrointestinal function and early mobilization.
- The main objectives of the ERAS program are to reduce the length of hospital stay after surgery and accelerate the return of patients to normal daily activities without increasing complications, hospital readmission rates and cost.
- The ERAS program has been adopted in several surgical specialties and is associated with faster and safer recovery, better quality of life and patient satisfaction.
- The process of implementing this program involves a multidisciplinary team and all units dealing with the surgical patient.
- Postoperative adverse events, venous thromboembolism is an example, are associated with longer hospital stay and higher mortality rates. Furthermore, factors such as postoperative pain and resumption of bowel function continue to be barriers to early discharge and return to daily activities.
- The program provides safe, high-quality perioperative care and should become standard practice for all women undergoing elective gynecological surgery.

## Recommendations

- Pre-admission counseling with information about the surgical procedure, anesthesia and postoperative care should be provided.
- Light meals can be taken up to six hours before surgery and clear liquids can be given up to two hours before surgery.
- Oral carbohydrates should be given two to three hours before induction of anesthesia.
- Bowel preparation should not be routinely performed.
- Perioperative thromboembolic prophylaxis should include dual-modality prophylaxis (heparin, pneumatic compression, and/or compression stockings) depending on the indication in each case.
- Intraoperative fluid overload should be avoided by adopting goal-directed therapy.
- Hypothermia should be avoided with intraoperative use of thermal blankets, circulating-water garments and warming of intravenous (IV) fluids.
- The use of drains, tubes and catheters should be avoided. If indispensable, their use should be limited to the shortest duration needed.
- Incisional infiltration with liposomal bupivacaine or bupivacaine should be incorporated into all ERAS protocols as a component of multimodal analgesia.
- Postoperatively, patients can drink immediately after surgery. Intravenous fluids should be discontinued when patients demonstrate ability to maintain oral hydration with at least 500 mL of oral fluid intake.
- Early mobilization and feeding should be encouraged.
- Multimodal opioid-sparing analgesia is recommended postoperatively, with greater emphasis on non-opioid drugs such as nonsteroidal anti-inflammatory drugs, acetaminophen, gabapentin, and dexamethasone.
- Multimodal approach to prevention and treatment of postoperative nausea and vomiting should be considered, with intraoperative use of at least two agents from different classes of antiemetics.

## Clinical context

The Enhanced Recovery After Surgery (ERAS – https://erassociety.org/ ) program represents a paradigm shift in conventional perioperative care, replacing, when necessary, some traditional practices with evidence-based practices and achieving better surgical quality, clinical improvements and lower costs to the health system. The program is based on perioperative optimization, including preoperative counseling, pain relief, carbohydrate intake, thromboembolism prophylaxis, standard anesthetic protocol, optimized fluid administration, recovery of normal gastrointestinal function, and early mobilization. 1

The ERAS program has been adopted in several surgical specialties and institutions around the world and was associated with a reduction in the average length of hospital stay and complication rates, in addition to a faster and safer recovery and improvement in quality of life and patient satisfaction. 1 An essential aspect for the implementation of an ERAS program is a multimodal and multidisciplinary approach. 2 The process of implementing this program involves a team composed of surgeons, anesthetists, an ERAS coordinator, nurses, nutritionists and physiotherapists of units that care for surgical patients. 3 Adherence to the program is crucial and the continuous auditing of the care process allows the team to have a comprehensive view of the patient's results ( Chart 1 ). 4

**Chart 1.** Principles of the ERAS program

Enhanced Recovery After Surgery (ERAS) Program		
What does it promote?	Why should it be implemented?	What is necessary for implementation?
- Minimization of the stress response of the operation, controlling perioperative physiology - Surgical medical optimization: preoperative counseling, pain relief, carbohydrate intake, thromboembolism prophylaxis, standard anesthetic protocol, optimized intraoperative fluid administration, recovery of normal gastrointestinal function and early mobilization	- Shorter hospital stay - No increase in rates of readmissions and/or reoperations and/or complications - Faster and safer patient recovery - Better quality of life and patient satisfaction - Reduction of general healthcare costs	- Program Coordinator - Involvement of all units that deal with the surgical patient - Multidisciplinary team working with the patient - Multimodal approach to solving problems that delay recovery and cause complications - Evidence-based scientific approach to care protocols - Change in management through interactive and continuous audits - Minimally invasive surgery whenever possible

Source: Adapted from Silva Filho AL, Santiago AE, Derchain SF, Carvalho JP. Enhanced Recovery After Surgery (ERAS): new concepts in the perioperative management of gynecologic surgery. Rev Bras Ginecol Obstet. 2018;40(8):433-6. doi: 10.1055/s-0038-1668581. 4

The main objectives of the ERAS program are to reduce the length of hospital stay after surgery and accelerate the return of patients to normal daily activities without increasing complications, hospital readmission rates or cost. 2 To this end, the ERAS program focuses mainly on minimizing the stress response of the operation, maintaining homeostasis, preventing catabolism with consequent loss of protein and muscle strength, in addition to minimizing cell dysfunction. 5

## What are the reasons for adopting the ERAS program in gynecological surgery?

Although most data are extrapolated from colorectal surgery, studies comparing the ERAS program to conventional practices in general gynecological surgery show positive results after implementation of the ERAS program, with a significant reduction in length of hospital stay, without an increase in readmission rates and complications in patients undergoing the practices recommended in the program. 1

Although most studies related to the ERAS program in gynecology have focused on open surgery, there is growing evidence of safety and feasibility for patients undergoing minimally invasive surgery, including intestinal procedures. 6 A publication by the Royal College of Obstetricians and Gynecologists reviewed the key elements of the ERAS program and suggested that it provides safe, high-quality perioperative care and should become standard practice for all women undergoing elective gynecological surgery. 7

Special focus is given to patients with gynecological cancer, since returning to the basal physiological level or close to it is essential for them, because it allows the performance of planned adjuvant therapies without delay, resulting in better oncological outcomes. 2 Non-randomized clinical trials involving patients with malignant gynecological neoplasms and the implementation of an ERAS program showed acceptable pain control, reduced hospital stay, adequate patient satisfaction and substantial cost reduction, with no difference in postoperative complications or mortality compared to conventional perioperative care. 8 This suggests the feasibility and safety of implementing the ERAS program in oncological gynecology, with benefits for patients undergoing major abdominal surgery. 4

## What to do before admission?

Pre-admission counseling is recommended with the aim to define expectations about surgical and anesthetic procedures and provide information about a care plan for the postoperative period. Preoperative education and psychological preparation can reduce anxiety and increase patient satisfaction, which can improve recovery and facilitate early discharge. 5

Health professionals and the nursing staff should identify the patient's expectations regarding hospitalization and show the benefits of early postoperative mobilization and feeding, postoperative pain control goals and length of hospital stay. 1 Guidelines such as the suspension of tobacco and alcohol use, which should occur four weeks before surgery, must be provided during this period. The offer of nutritional support also begins at this moment. 9

## What is the evidence on preoperative fasting and diet release after surgery?

Surgical stress after major surgery induces a marked and well-defined postoperative metabolic response. Using preoperative oral carbohydrates and avoiding prolonged preoperative fasting attenuates these postoperative responses. 5 Several randomized controlled trials have reported that clear fluids can be safely administered up to two hours and a light meal can be administered up to six hours before elective procedures that require general anesthesia ( Chart 2 ). 1 5 10

**Chart 2.** ERAS protocol: preoperative recommendations

Fasting: light meal up to six hours before and ingestion of clear liquids up to two hours before elective procedures that require general or regional anesthesia, or sedation/analgesia
Carbohydrate drinks *
Bowel preparation: routine use is not recommended in gynecological surgery
Preemptive analgesia: preoperative use of gabapentin, oral or intravenous cyclooxygenase 2 (COX-2) inhibitors (eg celecoxib) and oral or intravenous paracetamol
If higher risk of venous thromboembolism: dual mechanical prophylaxis (stockings and pneumatic compression) and chemoprophylaxis with low molecular weight heparin or unfractionated heparin

Source: Adapted from Kalogera E, Dowdy SC. Enhanced recovery pathway in gynecologic surgery: improving outcomes through evidence-based medicine. Obstet Gynecol Clin North Am. 2016;43(3):551-73. doi: 10.1016/j.ogc.2016.04.006. 1 Nelson G, Bakkum-Gamez J, Kalogera E, Glaser G, Altman A, Meyer LA, et al. Guidelines for perioperative care in gynecologic/oncology: Enhanced Recovery After Surgery (ERAS) Society recommendations-2019 update. Int J Gynecol Cancer. 2019;29(4):651-68. doi: 10.1136/ijgc-2019-000356. 5 Practice guidelines for preoperative fasting and the use of pharmacologic agents to reduce the risk of pulmonary aspiration: application to healthy patients undergoing elective procedures: an updated report by the American Society of Anesthesiologists Task Force on preoperative fasting and the use of pharmacologic agents to reduce the risk of pulmonary aspiration. Anesthesiology. 2017;126(3):376-93. doi: 10.1097/ALN.0000000000001452 10

* Attention to patients with delayed gastric emptying or gastrointestinal motility disorders – insufficient data for safety.

As for preoperative administration of oral carbohydrates two to three hours before induction of anesthesia, most protocols use a preoperative drink containing 50 g of carbohydrates. 5 11 In randomized controlled trials, oral carbohydrates have shown a better preoperative wellbeing, reduction of postoperative insulin resistance, decreased protein breakdown, maintenance of lean body mass and muscle strength, and provision of beneficial cardiac effects. 11 12

However, oral fluids, including oral carbohydrates, may not be safely administered in patients with delayed gastric emptying or gastrointestinal motility disorders, as well as in patients undergoing emergency surgery. 5 Although obese and diabetic patients have been included in recent studies with oral carbohydrates and no problems with regard to safety have been reported, the studies are insufficient to allow a general recommendation. 5 13

Maintaining an adequate nutritional status in the postoperative period leads to improvements in the return of intestinal activity, shorter length of hospital stay and lower rates of complications (for example, worse wound healing, anastomotic leaks or pulmonary complications). 5 14 Early feeding is considered the resumption of fluid and solid intake within 24 hours after surgery. According to the ERAS protocol, the patient is normally allowed to drink fluids upon recovery from anesthesia and is encouraged to resume the regular diet upon arrival in the room. Fundamental to the ERAS concept, oral ingestion is neither forced nor prohibited but encouraged, that is, the patient dictates the amount and type of oral ingestion. 1

There are currently no definitive guidelines for surgical patients regarding protein requirements, but a postoperative high-protein diet may reduce complications, and immunologic nutrition and arginine supplementation have shown promising results over regimens of heterogeneous nutrition. 5 15 16

## Should bowel preparation be performed?

Preoperative bowel preparation has traditionally been used to decrease postoperative infectious morbidity, including anastomotic leak after bowel surgery. Although this benefit has yet to be unequivocally proven, in addition to patient dissatisfaction, its use has been associated with preoperative dehydration and electrolyte abnormalities that can hamper postoperative recovery. Quality data from studies in colorectal surgery have shown that mechanical preparation alone does not reduce postoperative morbidity and should be abandoned. 5 More data are needed to guide the use of bowel preparation in elective rectal resections below the peritoneal reflection. 17

Bowel preparation with oral antibiotics may decrease infection rates in colorectal surgery, but high-quality evidence to support its use in gynecology is lacking. 1 Data from randomized clinical trials on the use of bowel preparation in gynecological surgery are limited to patients undergoing minimally invasive gynecological surgery. These studies conclusively showed that its use is not associated with better intraoperative visualization, ease of bowel handling or performance of procedures. 17 18 19

Surgeons who recommend bowel preparation should limit its use to patients with a colon resection planned. In such cases, the use of oral antibiotics alone should be considered or combined with mechanical bowel preparation. 5

## How to prevent thromboembolic complications?

Perioperative thromboembolic prophylaxis should include dual-modality prophylaxis and commence before induction of anesthesia. 20 The effectiveness of mechanical prophylaxis is equivalent to that of heparin alone and leads to a greater reduction in the risk of venous thromboembolism (VTE) when combined with heparin in gynecological cancer patients. Graduated compression stockings, when properly adjusted, also appear to decrease the rate of deep venous thrombosis in hospitalized patients, especially when combined with another method of prophylaxis for VTE. 5 The presence of malignancy, high body mass index, advanced age, pelvic surgery, extrapelvic disease, histology, use of preoperative corticosteroids, undergoing chemotherapy, immobility, and a hypercoagulable state have been identified as independent risk factors for VTE and are common among women undergoing gynecological surgery, especially for cancer. 21 All gynecological cancer patients undergoing major surgery lasting more than 30 minutes should receive dual mechanical VTE prophylaxis and chemoprophylaxis with low molecular weight heparin or unfractionated heparin, and dual prophylaxis should continue throughout the hospital stay. These patients meet the American College of Chest Physicians (ACCP) high-risk criteria and, for this reason, prolonged chemoprophylaxis for 28 days is recommended. 20 21

## How to maintain normothermia?

In the ERAS protocol, patient warming techniques are used since the preoperative period in order to minimize the initial drop in core temperature during anesthetic induction. These techniques, which include the intraoperative use of thermal blankets, circulating-water garment and warming of IV fluids, have shown to be effective in preventing hypothermia and should be continued throughout the surgery and in the post-anesthesia care unit. 22 Intraoperatively, continuous monitoring of core body temperature is essential to guide the management of these devices and prevent extreme body temperatures, including hypothermia and hyperthermia ( Chart 3 ). 1 5

**Chart 3.** ERAS protocol: intraoperative recommendations

Short-acting anesthetics: continuous infusion of propofol/short-acting opioid analgesics/total IV anesthesia with propofol/regional anesthesia with or without concomitant general anesthesia
Thermal blankets and IV fluid heating: continuous monitoring of core body temperature
Maintain euvolemia - goal-directed therapy: minimize the use of crystalloids and increase the use of colloids in the case of hypotension, although in the case of euvolemia, consider the use of vasopressors instead of liberal administration of crystalloids
Prevention of postoperative nausea and vomiting - intraoperative use of at least two antiemetic agents of different classes: 5HT3 antagonists (ondansetron), NK-1 antagonists (aprepitant), corticosteroids (dexamethasone), antihistamines (dimenhydrinate), anticholinergics (scopolamine), butyrophenones (haloperidol) and phenothiazines (chlorpromazine)
Limited use of drains, tubes and catheters. If indispensable, their use must be for the shortest time required

Source: Adapted from Kalogera E, Dowdy SC. Enhanced recovery pathway in gynecologic surgery: improving outcomes through evidence-based medicine. Obstet Gynecol Clin North Am. 2016;43(3):551-73. doi: 10.1016/j.ogc.2016.04.006. 1 Nelson G, Bakkum-Gamez J, Kalogera E, Glaser G, Altman A, Meyer LA, et al. Guidelines for perioperative care in gynecologic/oncology: Enhanced Recovery After Surgery (ERAS) Society recommendations-2019 update. Int J Gynecol Cancer. 2019;29(4):651-68. doi: 10.1136/ijgc-2019-000356. 5

Adequate control of body temperature is critical, because intraoperative body temperature below 36°C can lead to adverse intraoperative and postoperative outcomes, including coagulopathy, with higher risk of bleeding, impaired drug metabolism and oxygen transport, increased peripheral oxygen uptake, cardiac morbidity, and higher risk of surgical site infections. 23

## How to proceed with drains, tubes and probes?

The ERAS protocol recommends the limited use of drains, tubes and catheters, but if they are indispensable, the use should be limited to the shortest duration required. 1 Selective use or no use of nasogastric tube (NGT) was associated with earlier return of bowel function, less pulmonary complications, a trend toward shorter hospital length of stay, and no change in rates of anastomotic leak or other postoperative complications compared with routine NGT use. 5 9 In addition, the use of routine NGT has been associated with higher rates of postoperative pneumonia, atelectasis, and fever. 1

As for peritoneal drains, they should be considered in the ERAS protocol when there is a greater likelihood of postoperative pelvic collections, concerns about bleeding despite meticulous hemostasis, or very low intestinal resections without concomitant temporary intestinal deviation. 24 With the exception of intestinal anastomoses below the peritoneal reflection, where there may be a potential benefit in prophylactic drainage for a short period postoperatively, data do not support the routine use of prophylactic drainage after bowel resection. 1

Postoperatively, the recommendation is to remove urinary catheters within 24 hours after surgery, with some advocating removing them even earlier. 1 Studies have shown that patients who had the urinary catheter removed within the first few 24 hours after surgery showed less time to urinate spontaneously, with greater volume of urine and less need for re-catheterization for urinary retention, in addition to a shorter hospital stay. 25 When considering the intermediate removal (six hours after surgery), this seems superior to immediate removal (at the end of surgery) in terms of less frequent need for re-catheterization, and superior to late removal (within 24 hours postoperatively) in terms of less frequent urinary tract infections, earlier walking and shorter hospital stay. 26

## What is the recommended standard of anesthesia care?

Advances in anesthetic drugs and the expansion of outpatient care allowed the application of some of the principles of outpatient surgery to major surgery in order to mitigate the negative effects of surgical stress and pain, reduce side effects related to anesthetics and accelerate recovery. 1 Propofol has become the standard drug for induction of general anesthesia due to its rapid onset, favorable antiemetic profile, and rapid recovery. General anesthesia can be maintained with inhalation anesthesia or total IV anesthesia. 5 Short-acting inhalation agents such as sevoflurane or continuous infusion of propofol are recommended to allow rapid awakening from anesthesia, which is safely performed when these techniques are combined intraoperatively with short-acting opioid analgesics. Total IV anesthesia with propofol has been associated with fewer postoperative side effects and, specifically with a decrease in postoperative nausea and vomiting (PONV). 5 27

Regional anesthesia with or without concomitant general anesthesia has been associated with rapid awakening and decreased systemic opioid need. 5 Regional analgesic techniques include neuraxial anesthesia (eg, epidural, spinal), peripheral nerve blocks, and surgical wound infiltration. 28 Incisional infiltration with liposomal bupivacaine or bupivacaine has no systemic side effects when used properly and should be incorporated into all ERAS protocols as a component of multimodal analgesia. 1

## How to manage fluid administration?

Maintaining euvolemia is one of the principles of the ERAS program. 1 Fluid overload can lead to electrolyte abnormalities, peripheral soft tissue edema that impairs mobility, small bowel edema that contributes to the delay in the return of bowel function, and pulmonary congestion that leads to increased pulmonary morbidity. Hypovolemia, in turn, can result in decreased cardiac output, affecting the supply of oxygen to tissues, with consequent damage to organs. 29

To achieve intraoperative euvolemia, the ERAS protocol recommends avoiding intraoperative fluid overload, minimizing crystalloids and increasing the use of colloids. If a patient is hypotensive but at the same time euvolemic (which can occur after epidural anesthesia), the use of vasopressor rather than liberal administration of crystalloids is encouraged. 1 With this aim, some ERAS protocols have begun to adopt goal-directed therapy, a term used to describe the use of hemodynamic parameters such as stroke volume, cardiac output, peripheral vascular resistance, or similar parameters to guide the use of IV fluids and inotropic therapy. 30

Postoperatively, patients can drink fluids immediately after surgery, and IV fluids are stopped when they are able to maintain oral hydration (usually after they have ingested at least 500 mL of oral fluids). 1 Even in the immediate postoperative period, the rate of IV fluid administration is kept to a minimum, no more than 1.2 mL/kg, often much lower. In ERAS protocols, IV fluids are rarely needed beyond 12 to 24 hours postoperatively. 7 9 Balanced crystalloids (ringer lactate), which are solutions with an electrolyte concentration similar to that of plasma, are preferable to 0.9% saline solution for the prevention of hyperchloremic acidosis. 1

## How to optimize pain, nausea and vomiting control?

In the ERAS protocol, pain management begins before the incision. This theory is based on the concept of preemptive analgesia, in which analgesics block the activation of pain receptors before they are activated by the presence of noxious stimuli, resulting in superior pain control and decreased need for analgesics. A multimodal approach that incorporates the preoperative use of gabapentin, oral or IV cyclooxygenase 2 (COX-2) inhibitors (celecoxib or parecoxib), and oral or IV paracetamol has been associated with reduced postoperative opioid use, therefore, it is commonly used in ERAS protocols. 1

The use of opioids has traditionally been associated with increased PONV, compromised bowel function, delayed mobilization due to mental sensory changes, and increased pulmonary morbidity due to respiratory drive depression, in addition to a higher risk of dependence, leading to associated financial and social costs. 1 For these reasons, postoperative opioid-sparing multimodal analgesia is also recommended, with greater emphasis on non-opioid drugs such as non-steroidal anti-inflammatory drugs, acetaminophen, gabapentin and dexamethasone. The effectiveness of this approach is based on the synergistic action of two or more analgesics with different action mechanisms. 5

In addition to opioid-sparing analgesia, the ERAS protocol also adopts a multimodal approach for the preventive treatment of PONV, which includes intraoperative use of at least two agents from different classes of antiemetics. These classes of antiemetic drugs include 5HT3 antagonists, NK-1 antagonists, corticosteroids, antihistamines, anticholinergics, butyrophenones, and phenothiazines. Additional strategies to decrease PONV include the use of propofol infusion and less use of opioids. 31

## Why encourage early mobilization?

Early mobilization is a vital component of the ERAS protocol, as it protects against muscle and physical conditioning loss by avoiding prolonged bed rest and immobility. As a result, it helps to reduce pulmonary and venous thromboembolic complications, improves insulin resistance, and helps to reduce hospital stay. 1 In addition, early ambulation contributes to the return of bowel function, decreasing postoperative ileus rates. 5

## How to prevent postoperative ileus?

The return of bowel function is usually the last milestone reached before hospital discharge after a laparotomy. Among the factors influencing the return of bowel function are the use of opioids, the balance of venous fluids, the extent of peritoneal disease in the case of cancer patients, the complexity of surgery, the need for blood transfusion and postoperative abdominopelvic complications. 32

The implementation of minimally invasive surgery reduces the rate of postoperative ileus, but not all patients are candidates for this surgical approach. Among patients who need laparotomy, interventions that stimulate the enteric nervous system and reduce the use of opioids, such as early feeding, coffee consumption and chewing gum have shown to be effective in reducing the time for the return of bowel function in some studies. Although the use of chewing gum is safe and inexpensive, a large, recent, well-conducted randomized trial has shown no benefit. The consumption of coffee in the postoperative period has shown to reduce from 30% to 10% the rate of postoperative ileus in women undergoing gynecological cancer surgery. Furthermore, measures such as early ambulation and modal analgesia have shown a two to five times decrease of the rate of postoperative ileus. 5 33

The ERAS protocol also considers the possibility of using laxatives in order to accelerate the return of gastrointestinal function, since an earlier time for the first evacuation was observed when bowel stimulation with oral osmotic laxatives was performed within six hours after abdominal hysterectomy, with no change in pain and PONV scores. 1 34 As for prokinetics, there is little or no evidence to support their use for the purpose of preventing postoperative ileus ( Chart 4 ). 1 5

**Chart 4.** ERAS protocol: postoperative recommendations

Resumption of oral intake of liquids and solids within 24 hours after surgery: Encourage fluid intake when the patient recovers from anesthesia Consider high-protein diets
Early mobilization
Removal of the urinary catheter within 24 hours after surgery
Opioid-sparing multimodal pharmacological system for pain (two or more drugs) and regional analgesia: Combination of non-steroidal anti-inflammatory drugs (NSAIDs) with paracetamol Thoracic epidural analgesia, transverse abdominal blocks, wound infiltration with local anesthetic and intraperitoneal local anesthetic
If increased risk of venous thromboembolism: dual mechanical prophylaxis (stockings and pneumatic compression) and chemoprophylaxis with low molecular weight heparin or unfractionated heparin Extended chemoprophylaxis (28 days postoperative) for patients who meet high-risk criteria
Euvolemia: Discontinue IV fluids when patient can maintain oral hydration (at least 500 mL of oral fluids) In the immediate postoperative period, IV fluids should be maintained at a minimum not exceeding 1.2 mL/kg

## Final considerations

The principles of the ERAS protocol are applicable to all surgical specialties, and constant innovation must be the keynote to allow for the improvement of processes. The implementation of the ERAS program represents a paradigm shift in the perioperative management of surgical patients and is a multidisciplinary evidence-based approach. The program is clinically effective and impacts patient outcomes, providing a safe, high-quality approach and cost-effective perioperative care. In addition, a successful program can lead to faster and safer recovery and better quality of life and patient satisfaction. Therefore, the ERAS program should become standard practice for all women undergoing elective gynecological surgery.

National Specialized Commission on Ginecologic Endoscopy of the Brazilian Federation of Gynecology and Obstetrics Associations (FEBRASGO)

President:

Paulo Augusto Ayroza Galvão Ribeiro

Vice-President:

Mariano Tamura Vieira Gomes

Secretary:

Thomas Moscovitz

Membros:

Fabio Ohara

Francisco Eduardo Prota

Gustavo Anderman Silva Barison

Jean Pierre Barguil Brasileiro

Karin Kneipp Costa Rossi

Luciano Gibran

Luiz Flavio Cordeiro Fernandes

Raquel Papandreus Dibi

Raquel Silveira da Cunha Araújo

Ricardo Bassil Lasmar

Rita De Cássia Barbosa Tavares Santos

Romulo Muller dos Santos Melo

National Specialized Commission on Endometriosis of the Brazilian Federation of Gynecology and Obstetrics Associations (FEBRASGO)

President:

Julio Cesar Rosa e Silva

Vice-President:

Helizabet Salomao Abdalla

Secretary:

Márcia Mendonça Carneiro

Membros:

Carlos Alberto Petta

Carlos Augusto Pires Costa Lino

Corival Lisboa Alves de Castro

Eduardo Schor

João Nogueira Neto

João Sabino Lahorgue da Cunha Filho

Marco Aurélio Pinho de Oliveira

Marcos Tcherniakovsky

Maurício Simões Abrão

Omero Benedicto Poli Neto

Ricardo de Almeida Quintairos

Sidney Pearce Furtado

National Specialized Commission on Oncological Gynecology of the Brazilian Federation of Gynecology and Obstetrics Associations (FEBRASGO)

President:

Walquíria Quida Salles Pereira Primo

Vice-President:

Suzana Arenhart Pessini

Secretary:

Jesus Paula Carvalho

Membros:

Angélica Nogueira Rodrigues

Caetano da Silva Cardial

Delzio Salgado Bicalho

Eduardo Batista Candido

Etelvino de Souza Trindade

Fernando Maluf

Francisco José Cândido dos Reis

Georgia Fontes Cintra

Marcia Luiza Appel Binda

Mirian Helena Hoeschl Abreu Macedo

Renato Moretti Marques

Ricardo dos Reis

Sophie Françoise Mauricette Derchain

Heloisa de Andrade Carvalho

## References

[1] Kalogera E Dowdy S C Enhanced recovery pathway in gynecologic surgery: improving outcomes through evidence-based medicine Obstet Gynecol Clin North Am 2016 43 03 551 73 10.1016/j.ogc.2016.04.006 27521884

[2] Miralpeix E Nick A M Meyer L A Cata J Lasala J Mena G E A call for new standard of care in perioperative gynecologic oncology practice: impact of enhanced recovery after surgery (ERAS) programs Gynecol Oncol 2016 141 02 371 8 10.1016/j.ygyno.2016.02.019 26906066 PMC5989566

[3] Myriokefalitaki E Smith M Ahmed A S Implementation of enhanced recovery after surgery (ERAS) in gynaecological oncology Arch Gynecol Obstet 2016 294 01 137 43 10.1007/s00404-015-3934-4 26525694

[4] Silva Filho A L Santiago A E Derchain S F Carvalho J P Enhanced Recovery After Surgery (ERAS): new concepts in the perioperative management of gynecologic surgery Rev Bras Ginecol Obstet 2018 40 08 433 6 10.1055/s-0038-1668581 30142662 PMC10316925

[5] Nelson G Bakkum-Gamez J Kalogera E Glaser G Altman A Meyer L A Guidelines for perioperative care in gynecologic/oncology: Enhanced Recovery After Surgery (ERAS) Society recommendations-2019 update Int J Gynecol Cancer 2019 29 04 651 68 10.1136/ijgc-2019-000356 30877144

[6] Kalogera E Glaser G E Kumar A Dowdy S C Langstraat C L Enhanced recovery after minimally invasive gynecologic procedures with bowel surgery: a systematic review J Minim Invasive Gynecol 2019 26 02 288 98 10.1016/j.jmig.2018.10.016 30366117

[7] Torbé E Nordin A Acheson N Enhanced recovery in gynaecology Obstet Gynaecol 2013 15 04 263 8 10.1111/tog.12061

[8] Kalogera E Bakkum-Gamez J N Jankowski C J Trabuco E Lovely J K Dhanorker S Enhanced recovery in gynecologic surgery Obstet Gynecol 2013 122(2 Pt 1):319 28 10.1097/AOG.0b013e31829aa780 23969801 PMC3913481

[9] Nelson G Altman A D Nick A Meyer L A Ramirez P T Achtari C Guidelines for pre- and intra-operative care in gynecologic/oncology surgery: Enhanced Recovery After Surgery (ERAS®) Society recommendations – Part I Gynecol Oncol 2016 140 02 313 22 10.1016/j.ygyno.2015.11.015 26603969

[10] Practice guidelines for preoperative fasting and the use of pharmacologic agents to reduce the risk of pulmonary aspiration: application to healthy patients undergoing elective procedures: an updated report by the American Society of Anesthesiologists Task Force on preoperative fasting and the use of pharmacologic agents to reduce the risk of pulmonary aspiration Anesthesiology 2017 126 03 376 93 10.1097/ALN.0000000000001452 28045707

[11] Nygren J Thorell A Ljungqvist O Preoperative oral carbohydrate therapy Curr Opin Anaesthesiol 2015 28 03 364 9 10.1097/ACO.0000000000000192 25827282

[12] Azagury D E Ris F Pichard C Volonté F Karsegard L Huber O Does perioperative nutrition and oral carbohydrate load sustainably preserve muscle mass after bariatric surgery? A randomized control trial Surg Obes Relat Dis 2015 11 04 920 6 10.1016/j.soard.2014.10.016 25851776

[13] Laffin M R Li S Brisebois R Senior P A Wang H The use of a pre-operative carbohydrate drink in patients with diabetes mellitus: a prospective, non-inferiority, cohort study World J Surg 2018 42 07 1965 70 10.1007/s00268-017-4413-9 29282506

[14] Minig L Biffi R Zanagnolo V Attanasio A Beltrami C Bocciolone L Reduction of postoperative complication rate with the use of early oral feeding in gynecologic oncologic patients undergoing a major surgery: a randomized controlled trial Ann Surg Oncol 2009 16 11 3101 10 10.1245/s10434-009-0681-4 19760046

[15] McClave S A Taylor B E Martindale R G Warren M M Johnson D R Braunschweig C Guidelines for the provision and assessment of nutrition support therapy in the adult critically ill patient: Society of Critical Care Medicine (SCCM) and American Society for Parenteral and Enteral Nutrition (A.S.P.E.N.) JPEN J Parenter Enteral Nutr 2016 40 02 159 211 10.1177/0148607115621863 26773077

[16] Wischmeyer P E Carli F Evans D C Guilbert S Kozar R Pryor A American Society for Enhanced Recovery and Perioperative Quality Initiative Joint Consensus Statement on nutrition screening and therapy within a surgical enhanced recovery pathway Anesth Analg 2018 126 06 1883 95 10.1213/ANE.0000000000002743 29369092

[17] Fanning J Valea F A Perioperative bowel management for gynecologic surgery Am J Obstet Gynecol 2011 205 04 309 14 10.1016/j.ajog.2011.05.010 21704963

[18] Kantartzis K L Shepherd J P The use of mechanical bowel preparation in laparoscopic gynecologic surgery: a decision analysis Am J Obstet Gynecol 2015 213 05 7210 5 10.1016/j.ajog.2015.05.017 25981848

[19] Huang H Wang H He M Is mechanical bowel preparation still necessary for gynecologic laparoscopic surgery? A meta-analysis Asian J Endosc Surg 2015 8 02 171 9 10.1111/ases.12155 25384836

[20] Gould M K Garcia D A Wren S M Karanicolas P J Arcelus J I Heit J A Prevention of VTE in nonorthopedic surgical patients: Antithrombotic Therapy and Prevention of Thrombosis, 9th ed: American College of Chest Physicians Evidence-Based Clinical Practice Guidelines Chest 2012 141(2 Suppl):e227S e77S 10.1378/chest.11-2297 22315263 PMC3278061

[21] Lyman G H Khorana A A Kuderer N M Lee A Y Arcelus J I Balaban E P Venous thromboembolism prophylaxis and treatment in patients with cancer: American Society of Clinical Oncology clinical practice guideline update J Clin Oncol 2013 31 17 2189 204 10.1200/JCO.2013.49.1118 23669224

[22] Galvão C M Marck P B Sawada N O Clark A M A systematic review of the effectiveness of cutaneous warming systems to prevent hypothermia J Clin Nurs 2009 18 05 627 36 10.1111/j.1365-2702.2008.02668.x 19239533

[23] Scott E M Buckland R A systematic review of intraoperative warming to prevent postoperative complications AORN J 2006 83 05 1090 104, 107-13 10.1016/s0001-2092(06)60120-8 16722286

[24] Lopes A D Hall J R Monaghan J M Drainage following radical hysterectomy and pelvic lymphadenectomy: dogma or need? Obstet Gynecol 1995 86 06 960 3 10.1016/0029-7844(95)00311-e 7501348

[25] Griffiths R Fernandez R Strategies for the removal of short-term indwelling urethral catheters in adults Cochrane Database Syst Rev 2007 02 CD004011 10.1002/14651858.CD004011.pub3 17443536 PMC7163252

[26] Ahmed M R Sayed Ahmed W A Atwa K A Metwally L Timing of urinary catheter removal after uncomplicated total abdominal hysterectomy: a prospective randomized trial Eur J Obstet Gynecol Reprod Biol 2014 176 60 3 10.1016/j.ejogrb.2014.02.038 24670774

[27] Gupta A Stierer T Zuckerman R Sakima N Parker S D Fleisher L A Comparison of recovery profile after ambulatory anesthesia with propofol, isoflurane, sevoflurane and desflurane: a systematic review Anesth Analg 2004 98 03 632 41 10.1213/01.ane.0000103187.70627.57 14980911

[28] Wick E C Grant M C Wu C L Postoperative multimodal analgesia pain management with nonopioid analgesics and techniques: a review JAMA Surg 2017 152 07 691 7 10.1001/jamasurg.2017.0898 28564673

[29] MacKay G Fearon K McConnachie A Serpell M G Molloy R G O'Dwyer P J Randomized clinical trial of the effect of postoperative intravenous fluid restriction on recovery after elective colorectal surgery Br J Surg 2006 93 12 1469 74 10.1002/bjs.5593 17078116

[30] Gómez-Izquierdo J C Feldman L S Carli F Baldini G Meta-analysis of the effect of goal-directed therapy on bowel function after abdominal surgery Br J Surg 2015 102 06 577 89 10.1002/bjs.9747 25759947

[31] Gan T J Diemunsch P Habib A S Kovac A Kranke P Meyer T A Consensus guidelines for the management of postoperative nausea and vomiting Anesth Analg 2014 118 01 85 113 10.1213/ANE.0000000000000002 24356162

[32] Bakkum-Gamez J N Langstraat C L Martin J R Lemens M A Weaver A L Allensworth S Incidence of and risk factors for postoperative ileus in women undergoing primary staging and debulking for epithelial ovarian carcinoma Gynecol Oncol 2012 125 03 614 20 10.1016/j.ygyno.2012.02.027 22370599 PMC3909668

[33] American Society of Anesthesiologists Committee Practice guidelines for preoperative fasting and the use of pharmacologic agents to reduce the risk of pulmonary aspiration: application to healthy patients undergoing elective procedures: an updated report by the American Society of Anesthesiologists Committee on Standards and Practice Parameters Anesthesiology 2011 114 03 495 511 10.1097/ALN.0b013e3181fcbfd9 21307770

[34] Hansen C T Sørensen M Møller C Ottesen B Kehlet H Effect of laxatives on gastrointestinal functional recovery in fast-track hysterectomy: a double-blind, placebo-controlled randomized study Am J Obstet Gynecol 2007 196 04 3110 7 10.1016/j.ajog.2006.10.902 17403400

